# Current perspectives and emerging trends in iodine-125 seed implantation: a comprehensive bibliometric analysis

**DOI:** 10.1007/s11604-025-01805-6

**Published:** 2025-06-03

**Authors:** Zhao Liu, Qinghua Zhang, Xiancun Hou, Wei Shen, Yuan Zhu, Hui Zhu, Zhiyong Li

**Affiliations:** 1 Department of Nuclear Medicine, The Affiliated Hospital of Xuzhou Medical University, Xuzhou, 221006 China; 2 Cancer Institute, Xuzhou Medical University, Xuzhou, 221002 Jiangsu People’s Republic of China

**Keywords:** Iodine-125 seed, Brachytherapy, Neoplasms, Bibliometrics, Radiotherapy, VOSviewer

## Abstract

**Purpose:**

Iodine-125 (125-I) seed implantation is a widely used brachytherapy technique for treating various solid tumors. This study aims to provide a bibliometric analysis of the research trends, key contributors, and emerging hotspots in this field.

**Materials and methods:**

A comprehensive search was conducted using the Web of Science Core Collection, covering publications from January, 1960 to August 20, 2024. Bibliometric analysis was performed with VOSviewer, CiteSpace, and the R package “bibliometrix” to examine trends in publications, countries, institutions, journals, authors, and keywords.

**Results:**

The analysis included 2212 publications, showing a steady increase in research output, with USA, China, and Japan leading in publication volume. The University of California System and Keio University were the most productive institutions. *Brachytherapy* and the *International Journal of Radiation Oncology, Biology, and Physics* emerged as the most influential journals in this field. Yorozu Atsunori and Wang Junjie were identified as key authors. Keyword co-occurrence analysis highlighted “cancer,” “brachytherapy,” and “radiotherapy” as core themes. Keyword burst analysis revealed evolving research hotspots, such as “hepatocellular carcinoma,” “efficacy,” “safety,” and “transarterial chemoembolization,” emphasizing concerns about long-term outcomes, safety, and treatment strategies for 125-I implantation therapy across multiple cancers.

**Conclusion:**

This bibliometric analysis underscores that research on 125-I seed implantation is primarily focused on optimizing dosimetry, improving implantation techniques, and addressing long-term outcomes and safety. The findings emphasize the need for standardized treatment protocols to ensure consistent and effective clinical practice.

## Introduction

Iodine-125 (125-I) seed implantation, a form of low-dose-rate brachytherapy, has become a widely accepted method for treating solid tumors, including those in the prostate, lung, head and neck, and liver [1]. This technique delivers localized, continuous radiation directly to the tumor, minimizing damage to surrounding healthy tissues and reducing the risk of recurrence [2]. Over time, its clinical use has expanded significantly, leading to improved outcomes, lower toxicity levels, and enhanced patient quality of life. However, several challenges remain, including the optimization of treatment protocols, increasing the precision of seed placement, and addressing long-term complications [3].

In recent decades, research on 125-I seed implantation has grown substantially, contributing extensive data on its efficacy, safety, and innovations in delivery techniques [4]. Advances in imaging technologies, such as computed tomography (CT) and magnetic resonance imaging (MRI), have enabled more precise seed placement, enhancing treatment accuracy and outcomes [5]. Moreover, combination therapies incorporating 125-I seed implantation with external beam radiotherapy or chemotherapy have shown promise, particularly for aggressive tumors [6]. The integration of personalized treatment approaches, tailored to tumor characteristics and patient profiles, has further optimized therapeutic outcomes in brachytherapy [1].

Despite these advancements, the rapid expansion of research in this field has resulted in an overwhelming volume of publications, making it increasingly difficult for researchers, clinicians, and policymakers to stay informed about the latest developments. As the use of 125-I seed implantation expands beyond prostate cancer to include other tumors and palliative care applications [7], it has become crucial to systematically map the research landscape, identify key trends, and uncover emerging areas that may otherwise be overlooked. A bibliometric analysis offers a structured approach to achieving this, providing valuable insights into the historical progression, thematic developments, and potential future directions of research in this field.

Bibliometric analysis, which quantitatively examines academic literature, has proven effective in uncovering research trends and identifying priority areas [8, 9]. By analyzing patterns in publications, citations, and collaborations, such studies provide insights into influential work, key contributors, and evolving research areas [10, 11]. In recent years, bibliometric analyses have been widely applied in oncology, offering structured overviews of research activity in fields like external beam radiotherapy and proton therapy [12]. However, a comprehensive bibliometric evaluation focusing on 125-I seed implantation remains lacking, despite the growing body of literature on its clinical use. This study aims to fill this gap by conducting a bibliometric analysis of 125-I seed implantation research, highlighting thematic trends and potential future directions.

## Materials and methods

### Search strategies and data collection

We conducted a comprehensive literature search on the Web of Science Core Collection (WoSCC) [13]. The search formula was as follows [14, 15]: TS = (“iodine 125 seeds” OR “iodine-125 seeds” OR “iodine 125” OR “iodine-125”). The search covered publications from January 1960 to August 20, 2024. The starting date of January 1960 was chosen, because it represents the earliest documented use of 125-I in medical applications, providing a comprehensive historical perspective on the evolution of this treatment modality. The end date of August 20, 2024 ensured the inclusion of the most recent and up-to-date research available at the time of the analysis. Only articles written in English were considered.

### Statistical analysis

Data were extracted from the identified bibliographic records, and relevant bibliometric indicators were calculated using Microsoft Excel. These indicators included the number of annual publications, citation counts, average citations per article, journal names, journal impact factors, countries/regions of publication, affiliated institutions, and contributing authors.

For comprehensive visualization and analysis, three key bibliometric tools were employed: VOSviewer (version 1.6.20), CiteSpace (version 6.3.R1), and R package “bibliometrix” (https://bibliometric.com/). VOSviewer was instrumental in mapping institutional collaborations, author networks, co-authorship patterns, citation networks, and co-citation relationships [16]. It allowed us to visualize and explore complex relationships within the academic community, offering insights into the connections between authors, institutions, and publications. To identify emerging trends and research hotspots, we conducted a keyword co-occurrence analysis using VOSviewer and employed CiteSpace for keyword burst detection. CiteSpace was configured with the following parameters: time slicing from January 1994 to August 2024, with a yearly time interval, “Keyword” as the node type, and thresholds set to 5. The pruning strategy involved pathfinder pruning and merging networks to create a keyword timeline map. The R package “bibliometrix” was utilized to conduct a comprehensive bibliometric analysis.

The H-index was employed to quantify the academic impact of both individuals and journals. This metric reflects a researcher's productivity as well as the citation impact of their publications, thereby providing a balanced measure of academic influence. To assess the prestige and citation influence of journals, we utilized Journal Citation Reports (JCR) quartiles and Impact Factor (IF). JCR quartiles categorize journals into four tiers, with Q1 representing the highest level of academic impact. The IF quantifies the average number of citations received by articles published in a journal over the preceding two years. For this analysis, we employed the most recent 2023 release of JCR and IF data to ensure an up-to-date assessment of journal prestige and citation influence [17, 18].

## Results

### Publication trends

**Fig. 1:**
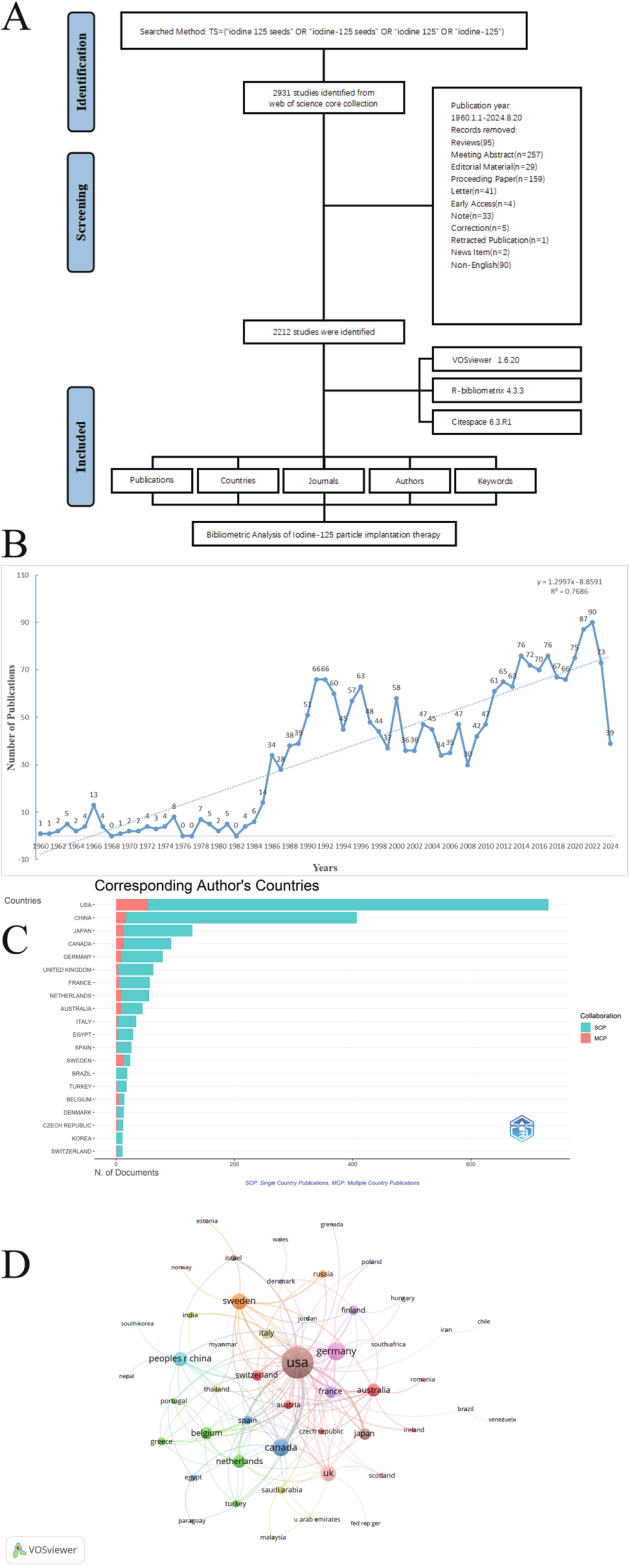
**A** Flowchart of data screening process. **B** Global research output and growth trends (1960–2024). **C** Distribution of publications by country. **D** International collaboration map

A detailed flowchart illustrating the literature screening process is presented in Fig. 1A. Between 1960 and 2024, 2212 publications related to 125-I seed implantation were identified. The overall publication trend showed significant growth, especially after the 1980 s, indicating a steady increase with an average annual growth rate of 5.89% (Fig. 1B).

### Country distribution of research and international collaborations

Between 1960 and 2024, a diverse array of countries contributed to research in 125-I seed implantation therapy (Table 1 and Fig. 1C). The USA emerged as the leading country in terms of both productivity and impact. It contributed 731 articles, accounting for 33.05% of the global output, and ranked first in total citations (TC) with 27,413 citations, averaging 37.5 citations per article. The USA also had the highest total publications (TP) at 1782 (TP rank = 1), further underscoring its dominant role in the field. China ranked second, with 407 articles (18.40% of the total output) and 4819 TC, averaging 11.8 citations per article. China's TP was 1072, ranking second (Table 1**)**. The analysis of international co-authorship revealed the patterns of global collaboration. Among the 62 countries involved in international collaborations, USA had the highest number of collaborations with other countries (144), followed by Germany (44) and Canada (40) **(**Fig. 1D**)**.

**Table 1:** Publication and citation profiles of leading countries

Country	Articles	Freq	MCP_Ratio	TP	TP_rank	TC	TC_rank	Average citations
USA	731	33.04702	7.250342	1782	1	27,413	1	37.5
China	407	18.39964	3.931204	1072	2	4819	2	11.8
Japan	129	5.831826	9.302326	485	3	1628	6	12.6
Canada	93	4.20434	12.90323	318	4	2256	3	24.3
Germany	79	3.571429	11.39241	226	6	1761	4	22.3
United Kingdom	63	2.848101	6.349206	181	8	1627	7	25.8
France	57	2.576854	8.77193	258	5	832	10	14.6
The Netherlands	56	2.531646	16.07143	167	9	1642	5	29.3
Australia	45	2.034358	20	196	7	881	8	19.6
Italy	34	1.537071	8.823529	145	10	555	12	16.3
Egypt	29	1.311031	6.896552	71	13	263	19	9.1
Spain	26	1.175407	3.846154	92	12	843	9	32.4
Sweden	24	1.084991	50	96	11	530	13	22.1
Brazil	19	0.858951	0	40	16	105	24	5.5
Turkey	18	0.813743	5.555556	38	17	176	21	9.8
Belgium	14	0.632911	35.71429	46	15	276	18	19.7
Denmark	13	0.587703	7.692308	32	19	340	15	26.2
Czech Republic	12	0.542495	16.66667	26	24	239	20	19.9
Korea	11	0.497288	0	29	20	310	17	28.2
Switzerland	11	0.497288	9.090909	47	14	816	11	74.2

Articles: publications of corresponding authors only

*Freq* frequency of total publications, *MCP_Ratio* proportion of multiple country publications, *TP* total publications, *TP_rank* rank of total publications, *TC* total citations, *TC_rank* rank of total citations, *Average citations* the average number of citations per publication

### Institutional contributions and collaborative networks

**Fig. 2:**
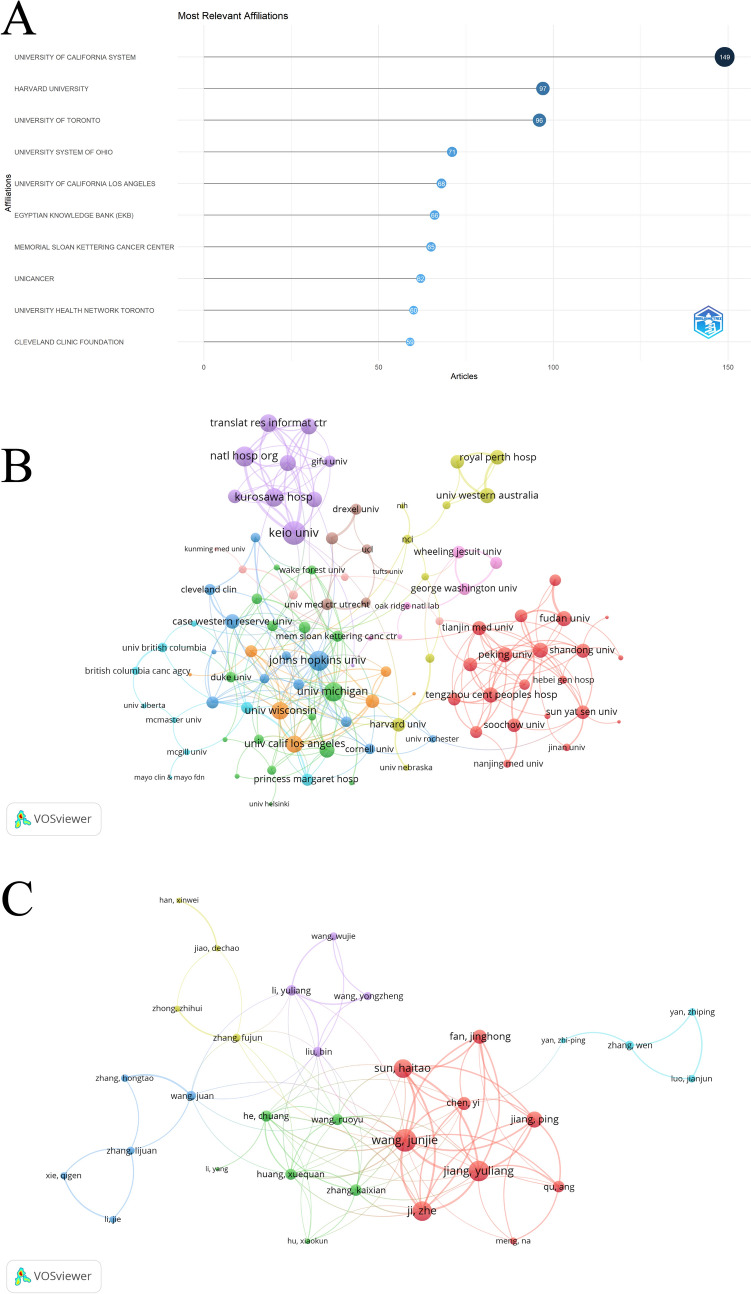
Analysis of institutions and author. **A** Top 10 institutions by article count. **B** Institutional collaboration map. **C** Influential authors and collaborative efforts

The top 10 most influential institutions in the field were identified, with the University of California System leading the way. It contributed the highest number of articles, with a total of 149 publications. Harvard University followed with 97 publications, while the University of Toronto ranked third with 96 publications (Fig. 2A). Among the 114 institutions involved in international collaborations, each with a minimum of 7 articles, Keio University had the highest number of collaborations with the other countries (40), followed by Johns Hopkins University and the National Hospital Organization, both with 29 collaborations (Fig. 2B).

### Analysis of influential authors and author networks

An analysis of high-impact authors in the field of 125-I particle implantation therapy identified 20 influential researchers, ranked by their H-index (Table 2). Adelstein SJ and Kassis AI shared the highest H-index of 17, with Adelstein publishing 19 articles (TP rank: 7) and Kassis publishing 20 articles (TP rank: 5). Among the top 20 authors, Yorozu Atsunori was the most productive, with 30 total publications (TP rank: 1) and an H-index of 12. Wang Junjie followed closely with 27 publications (TP rank: 2) and an H-index of 10. Saito Shiro, with 25 publications (TP rank: 3), also had an H-index of 12, matching Yorozu (Table 2).

**Table 2:** Publication and citation profiles of high-impact authors

Authors	H_index	g-index	m-index	PY_start	TP	TP_Frac	TP_rank	TC	TC_rank
ADELSTEIN SJ	17	19	0.34	1975	19	4.46	7	102	14
KASSIS AI	17	20	0.5	1991	20	5.30	5	88	20
SAITO SHIRO	12	18	0.631579	2006	25	2.85	3	100	15
YOROZU ATSUNORI	12	18	0.631579	2006	30	3.69	1	108	12
GUTIN PH	11	12	0.282051	1986	12	1.62	17	86	22
OHASHI TOSHIO	11	17	0.578947	2006	23	2.76	4	94	17
SHIELDS CAROL L	11	18	0.611111	2007	18	3.20	10	38	100
SNEED PK	11	11	0.323529	1991	11	1.47	21	75	27
LARSON DA	10	10	0.277778	1989	10	1.06	25	89	19
MCCANNEL TARA A	10	17	0.666667	2010	20	4.73	6	59	46
PHILLIPS TL	10	10	0.25641	1986	10	1.13	30	95	16
SHIELDS JERRY A	10	13	0.555556	2007	13	2.16	15	37	103
WANG JUNJIE	10	16	0.625	2009	27	3.42	2	104	13
JIANG PING	9	13	0.6	2010	13	1.65	14	64	41
JIANG YULIANG	9	12	0.642857	2011	19	2.38	8	62	42
SHIGEMATSU NAOYUKI	9	14	0.5	2007	19	2.29	9	66	37
TANAKA NOBUMICHI	9	11	0.5625	2009	11	0.97	22	40	86
WARA WM	9	9	0.25	1989	9	0.86	44	87	21
WEAVER KA	9	9	0.230769	1986	9	0.88	45	83	23
BRADY LW	8	9	0.222222	1989	9	1.35	32	48	62

*H_index* The h-index of the journal, which measures both the productivity and citation impact of the publications, *g_index* The g-index of the journal, which gives more weight to highly-cited articles, *m_index* The m-index of the journal, which is the h-index divided by the number of years since the first published paper, *TP* total publications, *TP_rank* rank of total publications, *TC* total citations, *TC_rank* rank of total citations, *Average citations* the average number of citations per publication, *PY_start* publication year start, indicating the year the journal started publication

Among the 144 authors involved in international collaborations (with a minimum of 6 articles), Yorozu Atsunori had the highest number of collaborations with other countries (170), followed by Saito Shiro (156) and Ohashi Toshio (137). Wang Junjie was also highly engaged in international collaboration, with 102 collaborations, often working closely with authors, such as Sun Haitao and Jiang Yuliang (Fig. 2C).

### Journal analysis and co-citation networks

The *International Journal of Radiation Oncology, Biology, and Physics* (IF = 6.4, Q1) had an H-index of 37, ranking second in TP (84 articles) and first in TC (4454 citations), reflecting its significant impact in the field. *Brachytherapy* (IF = 1.7, Q3) was the most prolific journal in 125-I seed implantation research, with 131 TP and ranking third in TC (1181 citations). The *Journal of Nuclear Medicine* (IF = 9.1, Q1) ranked fourth in TP (69 articles) and fifth in TC (1106 citations), while *Ophthalmology* (IF = 13.1, Q1) had the second-highest TP count (1,187 citations) despite publishing only 31 articles **(**Table 3**)**.

**Table 3:** Bibliometric indicators of high-impact journals

Journal	H_index	IF	JCR_Quartile	PY_start	TP	TP_rank	TC	TC_rank
International Journal of Radiation Oncology Biology Physics	37	6.4	Q1	1983	84	2	4454	1
Cancer	26	6.1	Q1	1984	39	6	663	11
Journal of Nuclear Medicine	26	9.1	Q1	1963	69	4	1106	5
Ophthalmology	23	13.1	Q1	1991	31	11	1187	2
Brachytherapy	21	1.7	Q3	2005	131	1	1181	3
Journal of Laboratory and Clinical Medicine	21	#N/A	#N/A	1985	69	3	#N/A	#N/A
Urology	21	2.1	Q2	1983	36	8	421	22
Archives of Ophthalmology	20	#N/A	#N/A	1987	27	15	858	6
American Journal of Obstetrics and Gynecology	17	8.7	Q1	1985	30	12	118	84
Medical Physics	16	3.2	Q1	1992	33	9	1162	4
Radiation Research	16	2.5	Q2	1971	25	16	643	12
European Journal of Nuclear Medicine	15	#N/A	#N/A	1991	29	13	1	4173
Radiotherapy and Oncology	15	4.9	Q1	1987	23	17	701	10
British Journal of Ophthalmology	12	3.7	Q1	1990	17	21	434	20
Radiology	12	12.1	Q1	1986	16	24	424	21
Science	12	44.7	Q1	1962	16	25	408	23
American Journal of Ophthalmology	11	4.1	Q1	1987	20	18	435	19
Applied Radiation and Isotopes	11	1.6	Q3	1988	32	10	226	37
Journal of Labelled Compounds & Radiopharmaceuticals	11	0.9	Q4	1987	38	7	195	50
Neurosurgery	11	3.9	Q1	1987	12	30	310	29

*H_index* the h-index of the journal, which measures both the productivity and citation impact of the publications, *IF* impact factor, indicating the average number of citations to recent articles published in the journal, *JCR_Quartile* the quartile ranking of the journal in the Journal Citation Reports, indicating the journal's ranking relative to others in the same field (Q1 top 25%, Q2 25–50%, Q3 50–75%, and Q4 bottom 25%), *TP* total publications, *TP_rank* rank of total publications, *TC* total citations, TC_rank rank of total citations, *Average Citations* the average number of citations per publication, *PY_start* publication year start, indicating the year the journal started publication

**Fig. 3:**
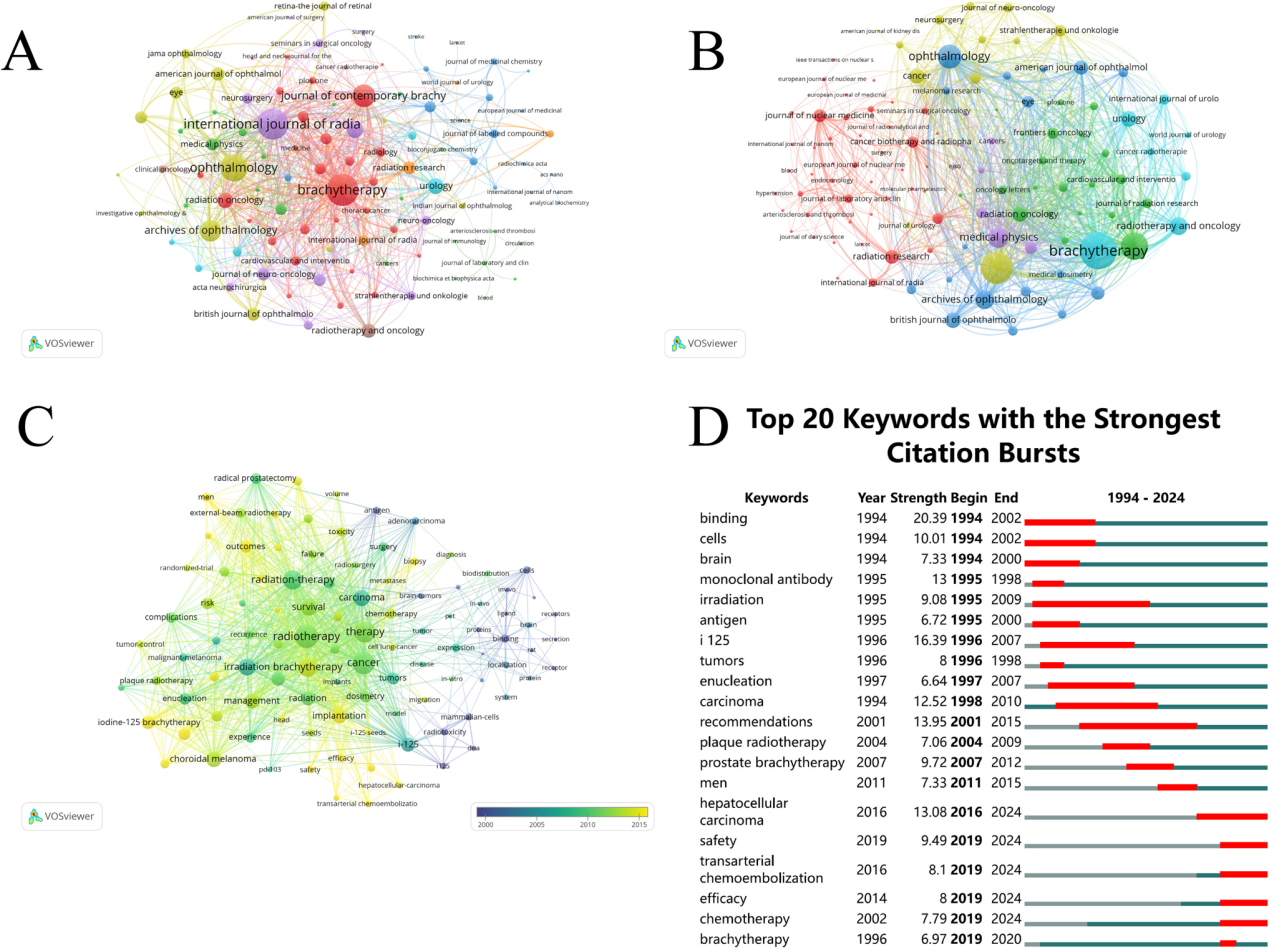
Analysis of journals and keywords. **A** Co-occurrence network of journals. **B** Citation coupling network of journals. **C** Keyword co-occurrence analysis. **D** Burst keyword analysis

The co-occurrence networks of journals included 114 journals with at least 4 occurrences. The three key journals with the highest total link strength in the co-occurrence networks were *Brachytherapy* (482), *International Journal of Radiation Oncology, Biology, and Physics* (448), and *Ophthalmology* (352) (Fig. 3A). The coupling networks of journals also included 114 journals, each with at least 4 couplings. The three key journals with the highest total link strength in the coupling networks were *Brachytherapy* (16,136), *International Journal of Radiation Oncology, Biology, and Physics* (12,001), and *Journal of Contemporary Brachytherapy* (8870) (Fig. 3B).

### Keyword co-occurrence and emerging research themes

The keyword co-occurrence network provides insights into how research on 125-I seed implantation therapy has evolved over time (Fig. 3C). The color coding in the network represents the timing of keyword appearance, with earlier research (2000 s) shown in blue and more recent research (2015 onward) in yellow. In the early 2000 s, keywords, such as “radiotherapy” and “cancer", were dominant, reflecting the focus on radiation treatments in oncology. As research progressed, terms like “brachytherapy” gained prominence, indicating the growing importance of brachytherapy techniques in cancer treatment (Fig. 3C).

In more recent years (indicated by the orange nodes), there has been a notable shift toward more specific technical and clinical aspects of 125-I seed implantation. Keywords, such as “implantation” and “dosimetry”, suggest an increased focus on the precision and safety of seed placement, as well as on the measurement and optimization of radiation dose delivery. The keyword “choroidal melanoma” also emerged frequently in the recent research, reflecting the growing use of 125-I brachytherapy for treating ocular tumors. Other keywords, including “management” and “outcomes”, highlight the continued emphasis on patient care and the evaluation of therapeutic efficacy (Fig. 3C).

The keyword burst analysis revealed significant shifts in research focus within the field over the past three decades (Fig. 3D). The strongest citation burst overall occurred for “binding” (strength: 20.39), which was prevalent between 1994 and 2002, reflecting early research interests in molecular interactions and biological mechanisms. In more recent years (2016–2024), the research focus has shifted toward optimizing treatment safety and efficacy, as well as exploring combination therapies. The keyword “hepatocellular carcinoma” (strength: 13.08, 2016–2024) highlights the growing application of 125-I brachytherapy in liver cancer treatments. Simultaneously, attention to treatment safety has increased, as evidenced by the prominence of “safety” (strength: 9.49, 2019–2024), reflecting efforts to mitigate risks associated with Iodine-125 therapies. The rise of “transarterial chemoembolization” (strength: 8.10, 2019–2024) and “chemotherapy” (strength: 7.79, 2019–2024) signals growing interest in integrating Iodine-125 brachytherapy with other therapeutic modalities to enhance treatment outcomes. Additionally, “efficacy” (strength: 8.00, 2019–2024) has emerged as a key keyword, underscoring the importance of evaluating and improving the effectiveness of these therapies. Throughout these phases, “brachytherapy” (strength: 6.97, 1996–2020) remained a constant keyword, reflecting the central role of this treatment method across different periods of research development (Fig. 3D).

## Discussion

The bibliometric analysis of 125-I seed implantation therapy reveals a rapidly growing research field, driven by advancements in brachytherapy and its applications across various tumor types. Our study analyzed 2212 publications from 1960 to 2024, uncovering key trends in publication growth, research collaboration, and evolving research themes. The results highlight the central role of countries like USA and China in driving research output, the importance of specific journals in disseminating high-impact studies, and the contributions of prominent authors who have significantly shaped the field.

125-I seed implantation has garnered increasing interest as a treatment modality due to several factors. Compared to external beam radiotherapy, brachytherapy offers the advantage of delivering a high dose of radiation directly to the tumor while minimizing exposure to surrounding healthy tissues, potentially leading to fewer side effects and improved quality of life [2, 19]. Furthermore, in certain cancers like prostate cancer, 125-I seed implantation has demonstrated comparable or superior outcomes to radical prostatectomy or external beam radiation, with potentially lower rates of impotence and incontinence [20]. For liver cancer, particularly hepatocellular carcinoma, 125-I seed implantation offers a minimally invasive option for patients who are not candidates for surgical resection or ablation, and can be combined with transarterial chemoembolization (TACE) to improve local tumor control [21, 22].

### National and institutional contributions

The geographical analysis revealed that USA, China, and Japan are the leading contributors to 125-I seed implantation research, with the University of California System and Harvard University being among the top institutions. The dominance of these countries can be attributed to their substantial investments in cancer research, advanced healthcare infrastructure, and strong funding mechanisms for radiotherapy innovations [1]. The USA, in particular, has historically led in cancer research and drug development, supported by institutions like the National Cancer Institute and the Food and Drug Administration, which play key roles in promoting clinical trials and new treatment modalities [23]. China’s rapid growth in this field is driven by its expanding healthcare system and increasing investments in cancer treatment technologies [2, 24, 25], which have led to a surge in domestic research output. In addition, collaborations between Chinese and international institutions have contributed to knowledge exchange and the adoption of advanced techniques in 125-I seed therapy [3]. Japan’s contributions, particularly through institutions like Keio University, are notable for their focus on precision medicine and international collaboration, which further strengthens their global research presence.

It is important to consider potential biases in the data based on country, institution, and author. For example, the increasing focus on hepatocellular carcinoma in recent years may reflect the higher prevalence of this cancer in certain regions, such as Asia, and the availability of 125-I seed implantation as a treatment option within those healthcare systems [21, 26]. Variations in medical insurance coverage and healthcare policies across different countries can also influence the adoption and research focus on specific treatments. Further studies are needed to investigate these potential biases and their impact on the overall research landscape of 125-I seed implantation.

In Japan, 125-I seed implantation is primarily utilized for the treatment of prostate cancer. The Japanese Prostate Permanent Seed Implantation Study Group (JPSS), comprising numerous institutions throughout Japan, plays a pivotal role in conducting prospective trials related to 125-I prostate cancer treatment [27]. This group has been instrumental in establishing standardized protocols and evaluating long-term outcomes of 125-I seed implantation for prostate cancer in the Japanese population. Notably, Dr. Saito Shiro and Dr. Yorozu Atsunori, who are identified in our analysis as key authors in the field, serve as representatives and vice representatives of JPSS, respectively, highlighting their significant contributions to the advancement of 125-I seed implantation in Japan [28].

### Keyword co-occurrence and keyword bursts

The keyword co-occurrence analysis in this study highlights the major themes and research directions in 125-I seed implantation therapy. The most frequently co-occurring keywords, such as “cancer,” “brachytherapy,” and “radiotherapy”, underscore the central role of 125-I seed therapy in cancer treatment, particularly in solid tumors like prostate cancer [20], liver cancer [21], and choroidal melanoma [29]. These results reinforce the well-established use of 125-I seeds in delivering localized radiation, a key characteristic of brachytherapy, which minimizes damage to surrounding healthy tissues while targeting tumor sites effectively [19].

Technical terms, such as “dosimetry,” “implantation,” and “management", were also frequently appeared in the co-occurrence network, indicating a continued focus on optimizing treatment protocols. Accurate dosimetry is fundamental to ensuring that the correct radiation dose is delivered to the tumor, thereby maximizing therapeutic efficacy and minimizing adverse effects [30]. The presence of “implantation” as a recurring keyword further emphasizes the importance of precise seed placement, which is critical for successful treatment outcomes, especially in anatomically complex regions like the liver and ocular structures [31].

This is particularly evident in their application to solid tumors, including prostate cancer, choroidal melanoma, liver cancer, and nasopharyngeal carcinoma. The frequent co-occurrence of “brachytherapy” and “radiation therapy” alongside these terms highlights the modality’s importance in delivering localized, low-dose-rate radiation, effectively minimizing damage to surrounding healthy tissues [32]. Moreover, the co-occurrence of “dosimetry” with “management” and “recommendations” suggests a growing interest in establishing standardized treatment protocols for 125-I seed implantation. This trend reflects the ongoing efforts to refine clinical guidelines, ensuring that the therapy is applied consistently and effectively across different tumor types. As the field expands beyond prostate cancer into more diverse applications, such as treating liver cancer and choroidal melanoma, the need for precise dosimetry and clear clinical guidelines becomes even more critical [26, 33].

The keyword burst analysis provides a dynamic view of how research trends in 125-I seed implantation have evolved over time. Early bursts, from 1994 through the early 2000 s, were dominated by foundational research keywords, such as “binding” (1994–2002) and “cells” (1994–2002), reflecting an initial focus on the biological mechanisms underlying radiation therapy. During this early period, research concentrated on understanding the interactions between radiation and tumor biology, while clinical applications were still being developed [34].

More recent bursts, from 2015 onwards, point to the expansion of 125-I seed therapy into new areas of application. Keywords like “hepatocellular carcinoma” (2016–2024) and “transarterial chemoembolization” (2019–2024) reflect an increasing focus on using 125-I seeds for liver cancer treatment, often in combination with other modalities like TACE. This suggests a growing interest in exploring combination therapies, particularly for difficult-to-treat cancers, where 125-I seed implantation may enhance the therapeutic effects of other treatments [35]. Additionally, the burst in keywords like “efficacy” (2019–2024) and “safety” (2019–2024) points to a continued focus on evaluating the long-term outcomes and safety profiles of 125-I seed therapy, especially as its use is broadened to include more complex and aggressive tumor types [22].

### Limitations

Several limitations are inherent in this bibliometric analysis of 125-I seed implantation therapy. First, although citation counts provide valuable insights into the academic impact of publications, they may not fully reflect the clinical relevance or practical applicability of the research. Articles with high citation counts may be influential within the academic community but may not necessarily translate into clinical advancements in 125-I seed implantation techniques. Second, the exclusion of non-English publications may limit the comprehensiveness of this study. Research published in languages other than English could offer significant insights into the global application and regional developments of 125-I seed implantation, particularly in countries where non-English literature is prevalent. Third, the search strategy was confined to WoSCC, which may have resulted in the omission of key studies that could provide a more complete understanding of 125-I seed implantation. While the primary focus of this bibliometric analysis was the clinical application of 125-I seeds in brachytherapy, some included studies focused on basic research, which may not directly relate to therapeutic applications. The inclusion of such studies could skew the representation of the field toward exploratory, non-clinical topics. Future analyses could benefit from refining search terms or applying stricter inclusion criteria to focus exclusively on clinical and therapeutic research.

## Conclusion

This bibliometric analysis offers a comprehensive overview of the evolving research landscape surrounding 125-I seed implantation, highlighting its expanding application beyond prostate cancer to include other malignancies, such as liver cancer and choroidal melanoma. Key areas of focus in the research include optimizing dosimetry, improving implantation techniques, and refining treatment protocols. These findings emphasize the need for standardized clinical guidelines to ensure consistent practice and enhance patient outcomes as the use of 125-I seed implantation continues to expand within oncological care.
